# Regions Enriched with Reverse Complement Triplets in Bacterial Genomes

**DOI:** 10.3390/ijms27052301

**Published:** 2026-02-28

**Authors:** Eugene V. Korotkov

**Affiliations:** Institute of Bioengineering, Research Center of Biotechnology of the Russian Academy of Sciences, Bld. 2, 33 Leninsky Ave., 119071 Moscow, Russia; katrin2@biengi.ac.ru; Tel.: +7-9267248271

**Keywords:** bacteria, genome, reverse complement triplets, sequence, method, DNA

## Abstract

I developed a mathematical method to search for DNA regions that are significantly enriched in reverse complement triplets (RCTs) and are located in sequences with strongly expressed triplet periodicity (TP). The method makes it possible to exclude the influence of TP on the number of RCTs. To search for RCTs, I used the difference between triplet frequencies and their expected number, which was determined by taking into account the TP of the analyzed region. I analyzed the genomes of 42 bacteria representing all bacterial phyla, and found that the number of DNA regions containing RCTs ranged from several hundred to several thousand per genome depending on its size. The average length of the region was about 850 DNA bases. The most common inversion symmetry (IS) pattern of the RCT-containing regions was the enrichment of the first, second, and third triplet positions with {A, G}, {A, T}, and {T, C} bases, respectively. When the sequence was rotated 180 degrees and the bases were replaced with complementary ones (IS), such enrichment of triplet positions was preserved. I suggest that the emergence of IS could be a result of evolutionary processes such as inversions, transpositions, and recombinations.

## 1. Introduction

Advances in sequencing technology have led to the accumulation of a large number of DNA and protein sequences and promoted the development of mathematical methods to identify genomic and proteomic structural patterns [1,2]. One such pattern is dispersed repeats (DRs), which are short repetitive nucleotide sequences distributed throughout genomes in a non-tandem, yet non-random, manner [3]. Typically, the degree of similarity between individual repeats ranges from 40 to 100%; often, DRs are associated with mobile elements such as transposons. Repeats constitute a significant part of eukaryotic genomes [4]; thus, 45% of the human genome consists of DRs [3].

Recently, we have developed a mathematical approach that allows statistically significant identification of DRs with similarity less than 40% and applied it to analyze bacterial genomes [5]. It appeared that DRs occupy from 17 to 72% of the bacterial genome and that most repeats are superimposed on coding sequences as a motif. In addition, the found DRs have inversion symmetry (IS) [5,6]. A detailed study of IS in the DRs of bacterial genomes shows that they display triplet periodicity (TP), which is characterized by matrix *M*(3,4) containing three columns and four rows that correspond to positions in the found DRs and to DNA bases, respectively [7,8]. Matrix *M* is calculated as multiple alignment *AL* constructed for the identified bacterial DRs. If the number of sequences in *AL* is *N*, then matrix *M* is filled as *m*(*i*,*s_k_*(*j*)) = *m*(*i*,*s_k_*(*j*)) + 1, where *i* = *j* − 3int(*j*/3) and *j* varies from 1 to *L* (*L* is the length of *AL* and *s_k_*(*i*) is the element of sequence *k* in *AL*). After matrix *M* is filled for all *k* from 1 to *N*, we obtain matrix *MM*(3,4) by first calculating sums $x(i)=\sum_{j=1}^{4}m(i,j)$, $y(j)=\sum_{i=1}^{3}m(i,j)$, $W=\sum_{i=1}^{3}\sum_{j=1}^{4}m(i,j)$, and *p*(*i*,*j*) = *x*(*i*)y(*j*)/*W*^2^, and then *mm*(*i*,*j*) = *m*(*i*,*j*) − *Wp*(*i*,*j*))/(*Wp*(*i*,*j*)(1 − *p*(*i*,*j*))^0.5^. Each cell *mm*(*i*,*j*) shows the argument of the normal distribution; the larger it is, the more the values of cell *m*(*i*,*j*) differ from those obtained for randomly shuffled sequences included in *AL*. It turned out that matrix *MM* for DRs of bacterial genomes has IS, which means that when all sequences in multiple alignment *AL* are rotated by 180 degrees and the bases are replaced with complementary ones, matrix *MM* looks the same because its values that are greater than 3.0 remain in the same cells. In 10 of the 12 studied bacterial genomes, these cells are 1G, 2A, 2T, and 3C (the number indicates the column and the letter—the row of matrix *MM*). In the other two genomes, the cells are 1G, 2C, 3A, and 3T [6]; such matrix *MM* also has IS but only with a phase shift: thus, if sequence GC(A/T)GC(A/T)GC(A/T)… is converted to sequence (T/A)GC(T/A)GC(T/A)GC…, the latter would result in the same matrix *MM* only if matrix *M* is filled from the second base.

It is very likely that the patterns found in [6] are a consequence of Chargaff’s second rule [9,10]. Applied to nucleotides and mononucleotides, it means that on a single DNA strand the frequency of nucleotide A is approximately equal to that of T and that of G to that of C. Chargaff’s generalized rule (also called IS) extends this principle to longer sequences [11,12], implying that the frequency of a *k*-mer is approximately equal to that of its reverse complementary (RC) *k*-mer. For example, according to this rule the frequencies of triplet AGC and its RC triplet GCT in a single DNA strand would be approximately equal. Such symmetry is observed in the genomes of most organisms, both eukaryotes and prokaryotes [13,14,15]; however, mitochondrial genomes often show significant deviations from this rule because of a different replication mechanism [16,17].

As discussed in [6], the formation of RC triplets (RCTs) in bacterial genomes may be, on the one hand, a result of evolutionary processes such as inversions, transpositions, and recombinations [18,19,20] and/or the formation of secondary DNA structures (e.g., hairpins) which requires intrachain complementarity [21,22]. On the other hand, RCTs may appear because of TP due to codon degeneracy. The uneven presence of amino acids in proteins, which results in the increased frequency of the encoding triplets, could also generate TP in DNA [7,23,24]. Thus, if a protein contains multiple serine and threonine residues encoded by AGT and ACT, respectively, the corresponding gene sequence should be enriched in two RCTs; a similar situation is observed with glycine and alanine encoded by GGC and GCC, respectively.

Mathematical methods used to search for RC *k*-mers usually operate based on *k*-mer frequencies [25,26,27]; however, in the presence of TP, RCT frequencies can be quite high merely on the account of nucleotide probabilities in the corresponding positions. For example, if a random sequence has *p*(1,a) = 0.4, *p*(1,t) = 0.4, *p*(2,c) = 0.4, *p*(2,g) = 0.4, *p*(3,a) = 0.4, and *p*(3,t) = 0.4, it would contain many ACT and TGA triplets; in this case, the occurrence of RCTs would be associated only with different base frequencies in the first, second, and third positions of the triplets and would be random in nature. Thus, the known methods to search for IS in the presence of TP find all RCTs irrespectively of their origin. However, it would be interesting to identify RCTs that have appeared exclusively through evolutionary processes or the assembly of secondary DNA structures. For this purpose, it is necessary to use a search parameter other than triplet frequency.

In this study, I developed a mathematical method for identifying DNA regions that are statistically significantly enriched in RCTs in the presence of strongly expressed TP. The advantage of the new method is that it allows excluding the influence of TP on the number of RCTs because it employs a new mathematical measure for RCT detection. The measure is the difference between triplet frequencies and their expected number, which is determined taking into account the TP of the analyzed region. I also defined some parameters of the introduced mathematical measure. The developed algorithm is applicable to search for DNA regions longer than 300 DNA bases. I used the method to analyze the genomes of 42 bacteria representing all bacterial phyla and found that the number of RCT-containing regions ranged from several hundred to several thousand per genome, depending on the genome size; the average region length was approximately 850 DNA bases. I then created RCT classes for each studied bacterium and RCT groups for all the 42 bacterial species. The *MM* matrix for the most common RCT group included cells 1/A, 1/G, 2/A, 2/T, 3/T, 3/C, and 3/G with the matrix values greater than 3.0 (6.7, 45.4, 46.6, 46.9, 7.3, 39.5, and 3.7, respectively). With the exception of cell 3G, all other cells converted to themselves upon reverse complement DNA transformation. I hypothesize that the emergence of RCTs is associated with evolutionary processes such as inversions, transpositions, and recombinations.

## 2. Results

### 2.1. Search for Regions Enriched with RCT in the Bacterial Genome

The genome sequence of *E. coli* strain K-12 substr. MG1655 (ASM584v2) was obtained from https://bacteria.ensembl.org/index.html (accessed on 20 February 2026). The search for RCT-containing regions in the *E. coli* genome was performed according to the algorithm described in Section 4.3. This search was performed on the website http://victoria.biengi.ac.ru/mct (accessed on 20 February 2026).

Table 1 shows the number of the identified sequences with RCTs depending on threshold *V*_0_ (Section 4.3). The results indicated that at *V*_0_ = 4.5, α = *N*_0_/*N_m_* ≈ 0.05. Therefore, I used *V*_0_ = 4.5 when searching for RCTs.

Let us consider an example of an RCT-containing region in the *E. coli* genome, which represents a sequence of 1200 bases (nucleotides 119,401–120,600) spanning the end of gene b0111 (119,281–120,135) and the beginning of gene b0112 (120,178–121,551), which encode AmpE and AroP proteins, respectively. For this sequence, *Vmax*(*a*) (Materials and Methods, Section 4.3) is equal to 5.7 and at *R*^1^ > 5.0 (Formula (1)) there are the following mirrored pairs of triplets: AAA/TTT, AAT/ATT, ATG/CAT, ACC/GGT, ACG/CGT, TAC/GTA, CAC/GTG, CAG/CTG, CGC/GCG, and GCC/GGC. For these triplet pairs, the $x_{T}^{1}(i)x_{MT}^{1}(i)$ values are 10.1, 16.5, 23.5, 9.8, 21.3, 9.5, 10.0, 90.4, 22.1 and 8.8, respectively, and the probability of $x_{T}^{1}(i)x_{MT}^{1}(i)$ > 5.0 for two normal distributions is less than 0.05.

I then searched for regions with RCTs in the genomes of bacteria from 42 phyla. All RCT-containing sequences identified in the bacterial genomes had a length that was a multiple of 3 bases. For each found sequence, I calculated $x_{T}^{0}(i)$ and $x_{MT}^{0}(i)$ as described for sequence *S* (Section 2.1). The use of *k* = 0 showed that I isolated triplets from the beginning of the sequence. I also calculated $R(i)=x_{T}^{0}(i)x_{MT}^{0}(i)$, which made it possible to determine pairs of triplets that formed RCT-enriched regions in the sequence because for them *R*(*i*) > 0. All sequences found in the bacterial genomes are presented in the Supplementary Materials, where for each bacterium, there is a directory named ‘Phyla’ including a directory named ‘Class’ which in turn contains the selection.txt file. In each of these files, the line beginning with KOK shows the number of the found sequence, KK and LL indicate the coordinates (beginning and end, respectively) of the sequence, lines starting with A=== and B=== show pairs of reverse complementary triplets, and Z and DL show the level of statistical significance *Vmax*(*a*) and the length of the sequence, respectively. The encoding (1 ≡ a, 2 ≡ t, 3 ≡ c, 4 ≡ g) is used; for example, triplet aaa is shown as 111. The field marked as RRR shows the *R*(*i*) values for the corresponding triplets, and the lines marked as CD1 and CD2 show the frequencies of triplets from sets *T* and *MT*. The threshold *V*_0_ was 4.5.

The data on the number of sequences with RCTs found in the 42 bacterial genomes are presented in Table 2. It can be seen that at *V*_0_ = 4.5, the rate of false positives was mostly within the interval from 1.6 to 21.5% (except *Bacillus anthracis* (42.7%) and *Candidatus poribacteria bacterium* (35.3%)) with an average of approximately 8%. This finding indicates the non-random presence of RCT regions in different bacterial genomes.

**Figure 1:**
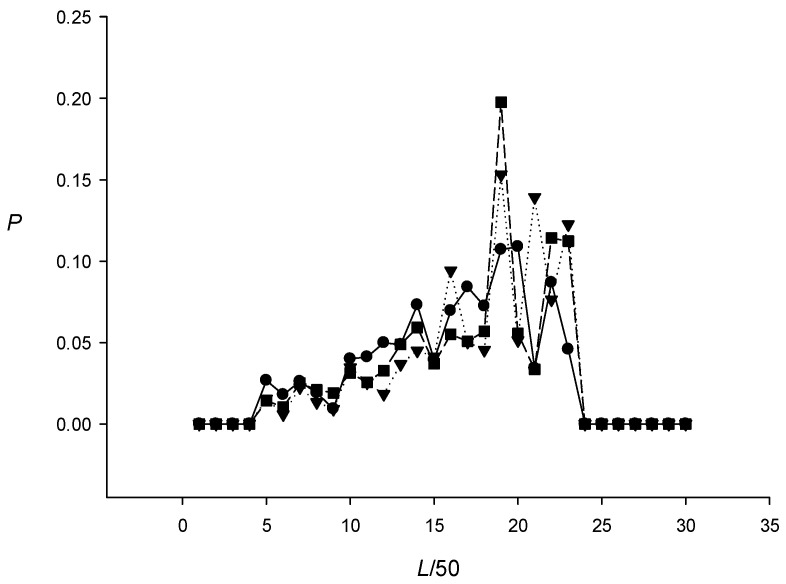
Length distribution for RCT-containing regions in the genomes of *E. coli* (circles), *Victivallis vadensis* (triangles), and *Gemmatimonas aurantiaca* (squares).

I analyzed the length distribution of the found RCT-enriched regions. The results for the genomes of *E. coli*, *Victivallis vadensis*, and *Gemmatimonas aurantiaca* are shown in Figure 1; similar results were obtained for the other bacterial genomes. The average length of a region containing RCTs ranged from 700 to 850 DNA bases.

### 2.2. Classes of RCT-Contaning Regions in the Bacterial Genomes

I created classes of sequences enriched with RCTs for each of the 42 bacteria, as described in Section 4.4. These results are summarized in the mirror.txt file consisting of directories for each bacterial phylum in Supplementary Materials. The file contains the following information. The lines beginning with SMAX and KMAX show the number of sequences with RCTs included in the created class and the central sequence number, respectively. The lines beginning with NOM contain the numbers of sequences from the selection.txt file that belong to the created class. The line beginning with KK contains the characteristics of the central sequence as in the selection.txt file (Section 4.3), including the coordinates (KK, LL), *V_max_* (*Z*), and length (DL). Lines beginning with *M* correspond to those in matrix *MM* (Section 4.4), and lines beginning with J contain the lines in matrix *A*(*i*,*j*) (Formula (9)). The lines beginning with A=== and B=== indicate pairs of reverse complementary triplets organized in a descending order of *RRR*(*i*) (Formula (11)).

From all mirror.txt files comprising RCT-enriched sequence classes for each bacterium, I created a single class_all.txt file in which the number of each bacterium is located after “start”, followed by a line titled “Phyla”. Then, from each matrix *A* of each class, I selected cells for which *A*(*i*,*j*) ≥ 3.0; the set of these cells shows the positions and bases with which the RCT-containing regions are enriched. Matrices *A* and *M* are included in the class_all1.txt file, and the set of positions in matrix *A* for which *A*(*i*,*j*) ≥ 3.0 is shown in the class_all.txt file in Supplementary Materials. For example, the first class from the *Acidobacterium capsulatum* genome contains 344 sequences (KOL = 344), and the set of positions with *A*(*i*,*j*) ≥ 3.0 are 1/T, 2/C, 2/G, and 3/A with values 19.6, 14.7, 15.3, and 20.9, respectively.

A total of 195 classes were created for the analyzed bacteria. For example, for the *E. coli* genome, six RCT-enriched classes containing 1549 sequences were created, which constitute about 90% of all sequences with RCTs found in the genome.

Table 3 shows that when inversion and complementary recoding were applied, positions 1/A, 1/C, 1/G, 2/A, 2/T, 3/T, 3/C, 3/G for which *A*(*i*,*j*) > 3.0 were preserved in triplets. The same happened in classes 2, 3, and 6. Classes 4 and 5 were also preserved though not completely; thus, position 1/C was lost in class 4 and 3/T, in class 5, but the remaining cells of the matrix were preserved.

### 2.3. Groups of RCT-Enriched Sequences for the Genomes of 42 Bacteria (Run_Class)

The groups of RCT-containing sequences created for different bacteria are included in the group100.txt file in Supplementary Materials. In this file, the line beginning with ‘Center’ contains the group number and the line beginning with ‘START’ incorporates the number of the central class and the number of classes included in the group (KOL(IM)). The next line (NAM) contains the name of the phyla where the central class was created; cells of this class, for which *A*(*i*,*j*) ≥ 3.0, are shown. Below, after ‘NAM1=’, the classes created in the class_all.txt file and included in the group are presented; each class begins with ‘K=’.

Table 4 shows the six groups of RCT-containing sequences from the 42 bacterial genomes. It can be seen that the first group comprised 77 classes and the central *MM* matrix was mirror-symmetrical with a complementary recoding of sequences, i.e., cells 1/A, 1/G, 2/A, 2/T, 3/T, and 3/C, where *A*(*i*,*j*) ≥ 3.0, did not change with recoding. There was only one exception, cell 3/G, but the matrix *A* value for this cell was quite small. All other groups shown in Table 4 had the same property.

### 2.4. Intersection of RCT-Enriched Sequences with Genomes, Mobile Elements, and Promoters

Intersection was considered if an RCT-enriched sequence was completely incorporated into the gene or, conversely, if the gene was completely incorporated into the RCT-enriched sequence. The number of such sequences was denoted as *N*. At the same time, I randomly shuffled the coordinates of the identified RCT-enriched sequences 200 times while maintaining their length and calculated the number of intersections for each shuffle, which was denoted as *N_r_*. Then, I calculated the mean and variance for *N_r_*, denoted as *D*(*N_r_*) and $\bar{N_{r}}$, respectively, and determined *Z* = (*N* − $\bar{N_{r}}$). The results showed that for all the bacteria in Table 2, *Z* ≤ 3.0, indicating that no significant correlations existed between the genes and RCT-enriched sequences. The same procedure was performed for promoter sequences, and, similarly, *Z* was ≤3.0 for all the bacteria from Table 2. Finally, I examined the intersection of the RCT-enriched sequences with mobile elements of the *E. coli* genome, which are listed in file eschrichia_coli_str_k_12_substr_mg1655_gca_000005845.gff3 from https://bacteria.ensembl.org/Escherichia_coli_str_k_12_substr_mg1655_gca_000005845/Info/Index/ (accessed on 20 February 2026). (for the other bacteria from Table 2, the coordinates of mobile elements are not specified). In this case, I were able to detect a correlation between the RCT-enriched sequences and the mobile element (Z = 4.0).

## 3. Discussion

In this work, I developed a new mathematical method for screening regions with IS in the genomes of bacteria. It has been shown that in bacterial genomes, approximately 80% are coding sequences characterized with TP [8] which can greatly influence triplet frequencies. My aim was to develop a mathematical method for finding regions with RCTs in the presence of TP. The results shown in Table 2 indicate that using my method, I could confidently identify RCT-containing regions in sequences with TP, as evidenced by a low rate of false positive results (8%) if random shuffling was performed with preservation of TP. It turns out that in addition to the factors leading to TP, there are those causing the formation of RCTs. Among them, evolutionary processes such as inversions, transpositions, and recombinations [18,19,20], as well as the generation of stem-loops [22], are the most instrumental in the emergence of RCTs; moreover, the binding of transcription factors could also promote RCT formation [13].

In my previous study, I have discovered RCTs in bacterial genomes during the search for DRs [6]. In 10 of the 12 examined bacterial species, regions enriched with RCTs have matrix *MM* for which cells 1G, 2A, 2T, and 3C contain values greater than 3.0; these fit completely into the first group of Table 4 here. In the other two species, matrix *MM* cells 1G, 2C, 3A, and 3T contain values greater than 3.0; such sequences belong to group 5 in Table 4 with a shift of one base (see Section 1). It can be stated that all RCT sequence classes found earlier were also identified in this work.

The method developed in this study is based on the approach to creating a sequence in which the formation of RCTs is associated with purely random factors (described in Section 4.2, step 4 and Section 4.3, step 7). In such a sequence, the correlations between adjacent DNA bases would be preserved but the formation of RCTs would be due to random factors. There are different ways to create such a sequence. First, an arbitrary sequence can be generated by random mixing of nucleotides. However, in this case *N_m_* values in Table 2 and Table 3 would be significantly overestimated because bacterial genomes consist of 80% coding sequences which have TP [7,23,24], and it would not be possible to understand whether I detect the regions with a significant number of RCTs or it is just a feature of TP.

Second, it is possible to shuffle nucleotide pairs instead of individual nucleotides. In this case, only some of the correlations between adjacent bases in the sequence would be preserved. For example, let us consider the first group in Table 4, in which cell G{A/T}C has the highest value of matrix *A*. There are two phases of base pair extraction for sequence. In the first phase, I obtain pairs G{A/T}, {A/T}C, and CG, and their random shuffling would produce a sequence containing combinations of these pairs, which means that in such a sequence RCTs can be partially preserved and their formation cannot be attributed to purely random factors. As a result, the level of *N*_0_ would be increased and that of *N_m_* decreased (Table 1). I verified this by randomly shuffling base pairs in the sequence in Section 4.2, step 4 and Section 4.3, step 7, which produced 1303 and 150 RCT-containing regions, respectively, for the *E. coli* genome (Table 1), indicating that this mixing method cannot be considered optimal.

Third, a scrambled sequence can be created using a Markov model constructed taking into account the observed correlations. However, this strategy is a mere expansion of the one described in the previous paragraph, and for such artificial sequences, the formation of RCTs also cannot be due to purely random factors.

In this study, my goal was to preserve the TP of the sequence which must be purely random. It was achieved by the method used in Section 4.2, step 4, and Section 4.3, step 7, when I split one nucleotide sequence into three according to the position in the triplet, then randomly shuffled each of the three sequences and reassembled the original one. Thus, this method preserves the TP of the sequence while detecting the regions with RCTs formed because of purely random factors.

**Figure 2:**
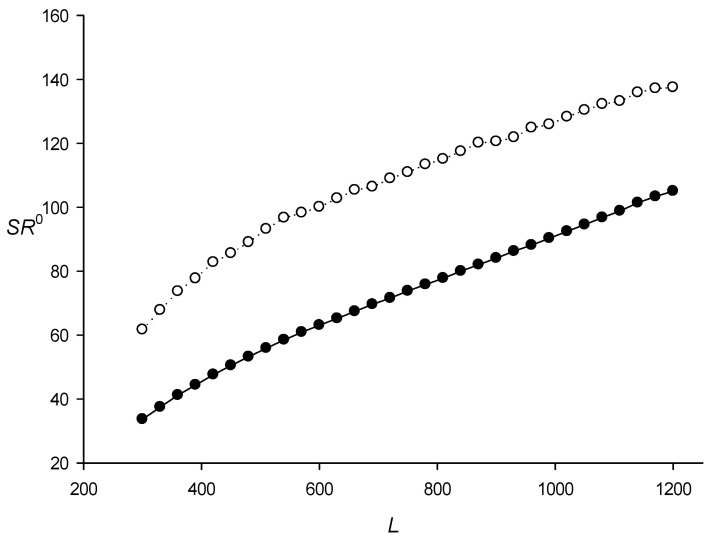
*SR*^0^(*L*,1) and *SR*^0^(*L*,4) depending on the length of sequence *S* from the *E. coli* genome (Section 4.2, step 9). Black and white circles indicate the first and fourth intervals, respectively.

**Figure 3:**
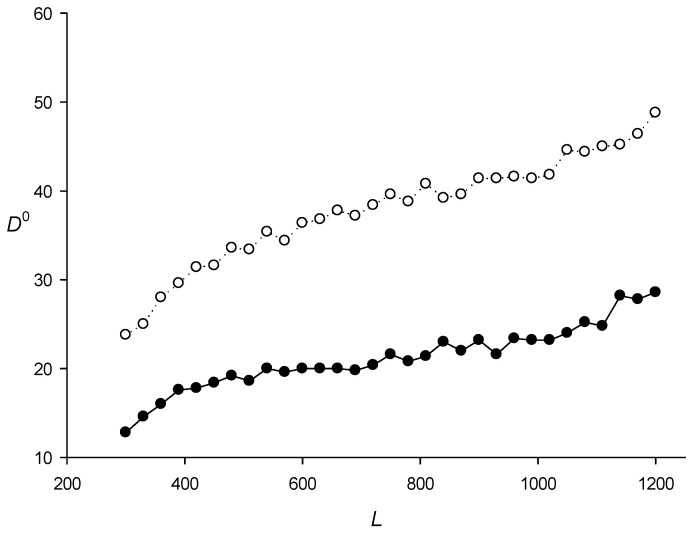
*D*^0^(*L*,1) and *D*^0^(*L*,4) depending on length *L* of sequence *S* from the *E. coli* genome (Section 4.2, step 9). Black and white circles indicate the first and fourth intervals, respectively.

It is also interesting to discuss the search for RCT-containing sequences in the genomes of 42 phylogenetically diverse bacteria (Table 2; here, *V*_0_ = 4.5 was chosen based on the *E. coli* genome analysis shown in Table 1). The results in Table 2 revealed that the ratio α = *N*_0_/*N*_m_ varied significantly: from 3.3% (*Helicobacter pylori*) to 42.7% (*Bacillus anthracis*), although most bacteria had α ≤ 10% and only few had large α values. Thus, the *V_max_*(*a*) distribution (Section 4.3, step 6) varies for different bacteria, which can lead to changes in *SR*^0^ and *D*^0^ (Section 4.2). Figure 2 and Figure 3 show *SR*^0^ and *D*^0^, which were calculated for the *E. coli* genome and were assumed to be the same for the other bacterial genomes. To test this assumption, I determined the *SR*^0^(*L*,*N*) and *D*^0^(*L*,*N*) spectra for different lengths *L* of the genomes of three bacteria. The results presented in Table 5 and Table 6 indicate that variations in *SR*^0^(*L*,*N*) and *D*^0^(*L*,*N*) did not exceed a few percent, which I thought was to be expected since only the sample size changed in the calculation of *SR*^0^(*L*,*N*) and *D*^0^(*L*,*N*).

I also analyzed the dependence of *SR*^0^(*L*,*N*) and *D*^0^(*L*,*N*) on the A + T content by comparing bacterial genomes with A + T from 32 to 74%. It turned out that there were variations in *SR*^0^(*L*,*N*) and *D*^0^(*L*,*N*) among the analyzed species (Table 5 and Table 6), which could result in different α values (Table 2). However, the average α for the 42 bacteria in Table 2 was 8% and such a comparatively low level of random noise cannot change the results shown in Table 3 and Table 4.

Previously, IS *k*-mers have been studied in complete genomes or in large sets of relatively short sequences. Thus, calculation of *k*-mer frequencies for 804 mitochondrial, 236 eubacterial, and 42 chloroplast genomes have been reported [17]. The advantage of the method described here is the possibility to find relatively short (from 300 to 1200 nucleotides) RCT-containing sequences and exclude the effect of TP, which allows attributing the nature of the found RCTs to evolutionary mechanisms.

## 4. Materials and Methods

### 4.1. Development of a Mathematical Parameter for Searching Sequences Enriched with Reverse Complementary Triplets

I took a bacterial genome of sequence *G* and length *L_G_*, and selected nucleotide sequence *S* within genome *G*, which has start coordinates equal to *a* and length *L*. Then, I created 64 triplets starting from position *a* + *k* (*k* can be 0, 1, and 2) to the end of sequence *S*, assuming that the first base of the first triplet was at base *k* of sequence *S* and the triplets were selected without overlap. The 64 triplets were divided into two equal groups so that each triplet in one group was reverse complementary to that in the other group. The first group was denoted as *T* = {aaa, aat, aac, aag, ata, atc, atg, aca, act, acc, acg, aga, agc, agg, taa, tac, tag, ttc, ttg, tca, tcc, tcg, tgc, tgg, cac, cag, ctc, ccc, ccg, cgc, gac, gcc} and the second as *MT* = {ttt, att, gtt, ctt, tat, gat, cat, tgt, agt, ggt, cgt, tct, gct, cct, tta, gta, cta, gaa, caa, tga, gga, cga, gca, cca, gtg, ctg, gag, ggg, cgg, gcg, gtc, ggc}. For each triplet in the two groups, I calculated $x_{T}^{k}(i)$ and $x_{MT}^{k}(i)$—the arguments of the normal distribution, which allow us to evaluate the abundance of the triplet in sequence *S*. To obtain $x_{T}^{k}(i)$ and $x_{MT}^{k}(i)$, I determined frequencies *f*(*i*) and probabilities *p*(*i*) = *f*(*i*)/*L* of bases in sequence *S*, where *i* is a DNA base. The probability of detecting a triplet from set *T* in sequence *S* was calculated as *P_T_*(*t*) = *p*(*i*)*p*(*j*)*p*(*k*), where *i*, *j*, and *k* are DNA bases located at the first, second, and third triplet positions, respectively. Then, the average number of triplets *t* in sequence *S* would be $\bar{n}(t)=(L/3)P_{T}(t),$ variance *D*(*t*) = (*L*/3)*P_T_*(*t*)(1 − *P_T_*(*t*)), and $x_{T}^{k}(i)=(n(t)-\bar{n}(t))/\sqrt{D(t)}$, where *n*(*t*) is the number of triplets *t* in sequence *S*. $x_{MT}^{k}(i)$ for set *MT* was calculated in the same way as $x_{T}^{k}(i)$ for set *T*. Finally, I calculated *R^k^*:

(1) $$R^{k}=\sum_{i=1}^{32}x_{T}^{k}(i)x_{MT}^{k}(i)$$

Each element of the sum $x_{T}^{k}(i)x_{MT}^{k}(i)$ is the product of two independent normal variables, the probability density of which can be described by a modified Bessel function of the second kind *K*_0_ [28]. The larger the value of *K*_0_, the lower the probability that $x_{T}^{k}(i)$ and $x_{MT}^{k}(i)$ are independent of each other. For this reason, it is convenient to take *R^k^* as a measure of the independence of $x_{T}^{k}(i)$ and $x_{MT}^{k}(i)$ and for all *i* from 1 to 32.

### 4.2. Determination of Mean Values SR^k^(L,N) and Variance D^k^(L,N) for R^k^

If I assume that sequence *S* is purely random, then each product $x_{T}^{k}(i)x_{MT}^{k}(i)$ for *i* from 1 to 32 has normal product distribution [27]. However, bacterial genomes cannot be considered purely random sequences, as there is strong correlation between neighboring DNA bases, which can be observed as TP [8] and which can significantly change distributions *R^k^* (*k* = 0, 1, 2). Therefore, I determined average value *SR^k^*(*L*,*N*) and variance *D^k^*(*L*,*N*) for *R^k^* depending on TP values and *L* of sequence *S*.

To calculate TP, I created periodic sequence *S*_1_ of length *L*, which contained numbers 123123123…, and filled matrix *M*(3,4) as *M*(*s*_1_(*i*),*s*(*i*)) = *M*(*s*_1_(*i*),*s*(*i*)) + 1, where *i* varies from 1 to *L*. Mutual information *I* [29] was determined using the following formula:

(2) $$I=\sum_{i=1}^{3}\sum_{j=1}^{4}m(i,j)\ln m(i,j)-\sum_{i=1}^{3}x(i)\ln x(i)-\sum_{j=1}^{4}y(j)\ln y(j)+L\ln L$$

where $x(i)=\sum_{j=1}^{4}m(i,j)$ and $y(j)=\sum_{i=1}^{3}m(i,j)$, and the argument of normal distribution *Z* was calculated as

*Z* = (4*I*)^0.5^ − (11.0)^0.5^ (3)

*Z* ranges from −3.0 to +3.0 for sequences with no significant correlation between adjacent bases but increases with the increase in the correlation. To take this into account in the calculation of *R^k^* (*k* = 0, 1, 2), I introduced seven intervals *N* for *Z* (Table 7). The intervals were chosen to minimize the number of false positives when searching for RCT-enriched regions in the *E. coli* genome; each interval also contained a sufficient number of regions to calculate mean value *SR^k^*(*L*,*N*) and variance *D^k^*(*L*,*N*), which were necessary to introduce a quantitative parameter indicating statistically significant presence of RCTs in the sequence. I used the deviation of *R^k^* from the mean divided by the square root of the variance as such a parameter. *SR^k^*(*L*,*N*) and *D^k^*(*L*,*N*) were calculated according to the Algorithm 1.
**Algorithm 1.** The algorithm for calculating *SR^k^*(*L*,*N*) and *D^k^*(*L*,*N*) is shown hereStep 1Let us introduce array *SP^k^*(*L*,*N*,*J*) and set its initial values to zero. In total, I considered 31 intervals for *L* (from 300 to 1200 bases with a step of 30) and 7 intervals *N* for *Z*; *J* was the ordinal number.Step 2First, I chose *k* = 0, *J* = 0, and *L* = 300 bases. Subsequently, I used *L* values that were multiples of three bases.Step 3Sequence *S* was taken from the genome of bacterium *G* starting from base *a* + *k*.Step 4I calculated *J* = *J* + 1 and shuffled sequence *S* while preserving TP in order to maintain the correlations between DNA bases among randomly selected triplets. To do this, I created three sequences, *S*_1_, *S*_2_, and *S*_3_, where bases were selected according to their number in sequence *S*; these sequences contained bases located at positions 3*n* + 1, 3*n* + 2, and 3*n* + 3 of sequence S, respectively (here, *n* = 1, 2, 3… as long as 3*n* + 3 < *L*). Then, each of the three sequences was randomly shuffled and recombined into a single sequence *S*. Thus, the formation of RCTs could be attributed only to random factors.Step 5*R^k^* was calculated, and interval *N* corresponding to *Z* was determined from Table 7. Then, *SP^k^*(*L*,*N*,*J*) = *R^k^*.Step 6Sequence *S* was shifted by *L* bases from the beginning of the genome (*a* = *a* + *L*), and I returned to step 4. However, if *J* > int(*L_G_*/*L*) (*L_G_* is the length of the bacterial genome), then the end of the bacterial genome was reached, and I proceeded to step 7.Step 7I added 30 bases to *L*, set *J* to 0, and went back to step 3. If *L* > 1200, I proceeded to step 8.Step 8I took *k* = *k* + 1 and went back to step 1. If *k* > 2, I proceeded to step 9.Step 9I calculated *SR^k^*(*L*,*N*) and *D^k^*(*L*,*N*), which are the mean and variance, respectively, of *R^k^* for each interval *Z* (Table 7) and all values of *L*.

**Figure 4:**
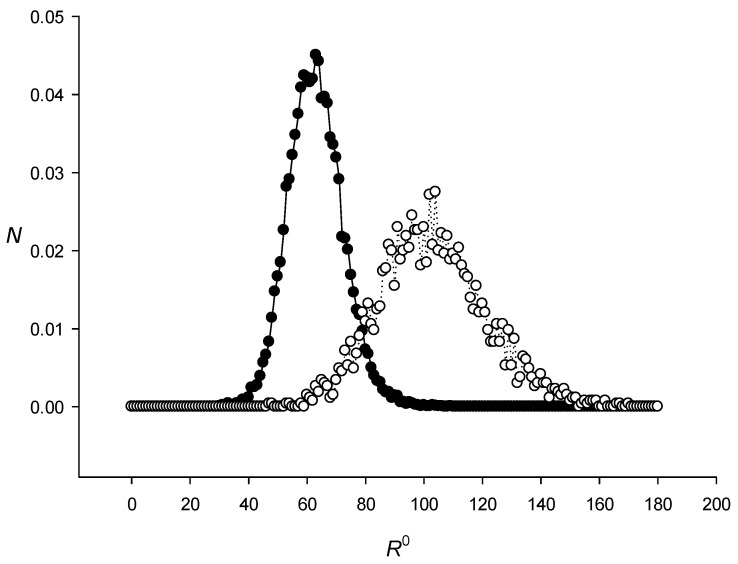
The distribution of *SP*^0^(600,1,*J*)/*Sum*_1_ (black circle) and *SP*^0^(600,4,*J*)/*Sum*_2_ (white circle) for all *J* (step 6 above) that fall within the interval from *R*^0^ to *R*^0^ + 1 in the *R*^0^ range from 0 to 200. These quantities are shown in the figure as *N*. *Sum*_1_ and *Sum*_2_ are the sum of *SP*^0^(600,1,*J*) and *SP*^0^(600,4,*J*) for all *J*, respectively. *R*^0^ shows the intervals for *SP*^0^(600,1,*J*) and *SP*^0^(600,4,*J*).

As an example, Figure 4 shows the distribution of *SP*^0^(600,1,*J*)/*Sum*_1_ and *SP*^0^(600,4,*J*)/*Sum*_2_ for all *J* (step 6 above) that fall within the interval from *R*^0^ to *R*^0^ + 1 in the *R*^0^ range from 0 to 200. These quantities is shown in the figure as *N*. *Sum*_1_ and *Sum*_2_ are the sum of *SP*^0^(600,1,*J*) and *SP*^0^(600,4,*J*) for all *J*, respectively. It can be seen that *R^k^* strongly depended on the TP level in sequence *S*. Figure 2 shows *SR*^0^(*L*,1) and *SR*^0^(*L*,4) and Figure 3—*D*^0^(*L*,1) and *D*^0^(*L*,4) obtained for the complete *E. coli* genome. The results indicated that *SR^k^*(*L*,*N*) and *D^k^*(*L*,*N*) did not depend on *k*, i.e., *SR^k^*(*L*,*N*) and *D^k^*(*L*,*N*) were equal within the statistical error for all three *k* values. Full results of the calculations of *SR^k^*(*L*,*N*) and *D^k^*(*L*,*N*) are presented in the sred.txt file.

### 4.3. Searching the E. coli Genome for Sequences Enriched in RCTs

As mentioned in Section 4.1, *a* indicates the starting coordinate of sequence *S* in the *E. coli* genome. To search for DNA sequences enriched in RCTs, I used $V_{m}^{k}$, which is the maximum of *V^k^*(*l*_1_,*l*_2_) for all values of coordinates *l*_1_ and *l*_2_ of subsequence *Seq^k^*(*l*_1_ + *k*,*l*_2_) in sequence *S*. *V^k^*(*l*_1_,*l*_2_) was calculated as

*V^k^*(*l*_1_,*l*_2_) = {*R^k^*(*l*_1_,*l*_2_) − *SR^k^*(*l*_2_ − *l*_1_ + 1,*N*)}/{*D^k^*(*L*,*N*)^0.5^} (4)

where *k* can take values 0, 1, and 2, and *N* is the number of the interval from Table 7, into which the TP sequence in coordinates from *l*_1_ to *l*_2_ falls.

**Figure 5:**
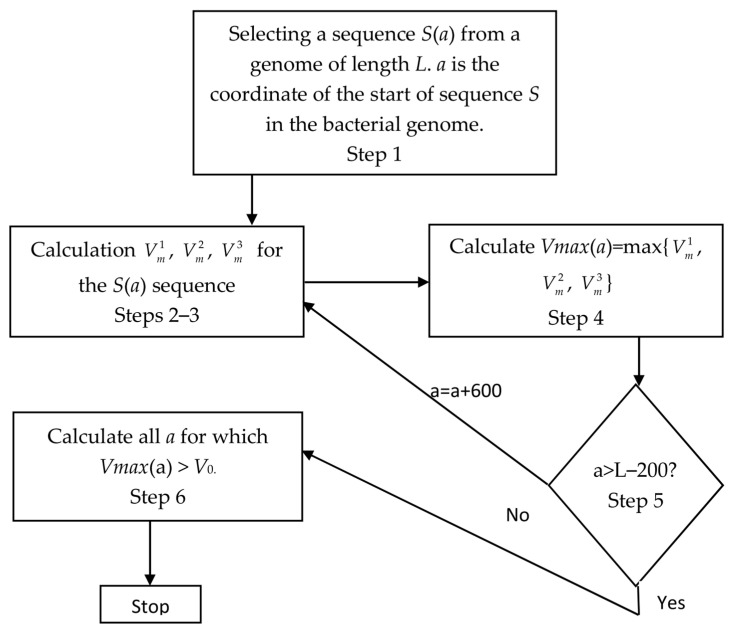
A graphical diagram of the Algorithm 2 for finding sequences enriched in RCTs is shown in this figure. A detailed description of the algorithm is shown in Section 4.3.

The calculations were performed using Algorithm 2. The graphical diagram of the Algorithm 2 is shown in Figure 5.
**Algorithm 2.** Here is an algorithm that was used to search for DNA sequences enriched in RCTsStep 1Sequence *S* with a length of 1200 bases was selected from the beginning of the *E. coli* genome starting from *a*.Step 2Subsequence *Seq^k^*(*l*_1_,*l*_2_) was selected in sequence *S*. *k* was initially equal to 0, coordinate *l*_1_ varied from base *k* + 1 to base 900 + *k* of sequence *S*, and coordinate *l*_2_ varied from *l*_1_ + 300 to 1200 with a step of 30 bases. Mutual information *I* was calculated for each sequence *Seq^k^*(*l*_1_,*l*_2_) using Formula (2), and *Z*(*l*_1_,*l*_2_) was calculated using Formula (3) (Section 4.2). Then, interval number *N* was determined for *Z*(*l*_1_,*l*_2_) in Table 7 and used to calculate *R^k^* according to Formula (1), which allowed determination of *V*(*l*_1_,*l*_2_) for sequence *Seq^k^*(*l*_1_,*l*_2_) according to Formula (4).Step 3I selected coordinates *l*_1_, *l*_2_ for which the maximum value of *V^k^*(*l*_1_,*l*_2_) was achieved; these coordinates were termed *l*_1*m*_, *l*_2*m*_ and the maximum $V_{m}^{k}$. Then, *k* = *k* + 1 and all calculations were redone starting from step 2. If *k* > 2, I proceeded to step 4.Step 4As a result, I obtained three values ($V_{m}^{1},V_{m}^{2},V_{m}^{3}$) and selected the largest of them denoted as *V_max_*(*a*), whereas the corresponding *k* was denoted as *k_max_*.Step 5I took *a* = *a* + 600 and proceeded to step 1. If *a* was greater than *L_G_* − 1200, I proceeded to step 6.Step 6As a result of the calculations, I obtained vector *V_max_*(*a*) where *a* varied from 1 to *L_G_*-1200. Then, I entered a threshold value for *V_max_*(*a*) denoted as *V*_0_ and selected all *a* where *V_max_*(*a*) > *V*_0_; the number of these values was denoted as *N_m_*.Step 7All the calculations in steps 1–6 were repeated with the following changes. In step 2, I randomly shuffled *Seq^k^*(*l*_1_,*l*_2_) as I did above in the calculation of *SR^k^*(*L*,*N*) and dispersion *D^k^*(*L*,*N*) (Section 2.2, step 4). During such shuffling, triplets were changed but TP was preserved. In this case, *Z*(*l*_1_,*l*_2_) was influenced only by the correlation of bases and the number of RCTs was related solely to random factors. Here, I also selected all *a* where *V_max_*(*a*) > *V*_0_ and denoted the number of such values as *N*_0_.

Then, I calculated α = *N*_0_/*N_m_*. As a result, I obtained the values of *V_max_*(*a*), number of sequences *N_m_* enriched with RCTs, the coordinates of the found sequences (*a*, *l*_1*m*_, *l*_2*m*_), and *k_max_*.

### 4.4. Classification of Regions Enriched with RCTs in the Bacterial Genome

**Figure 6:**
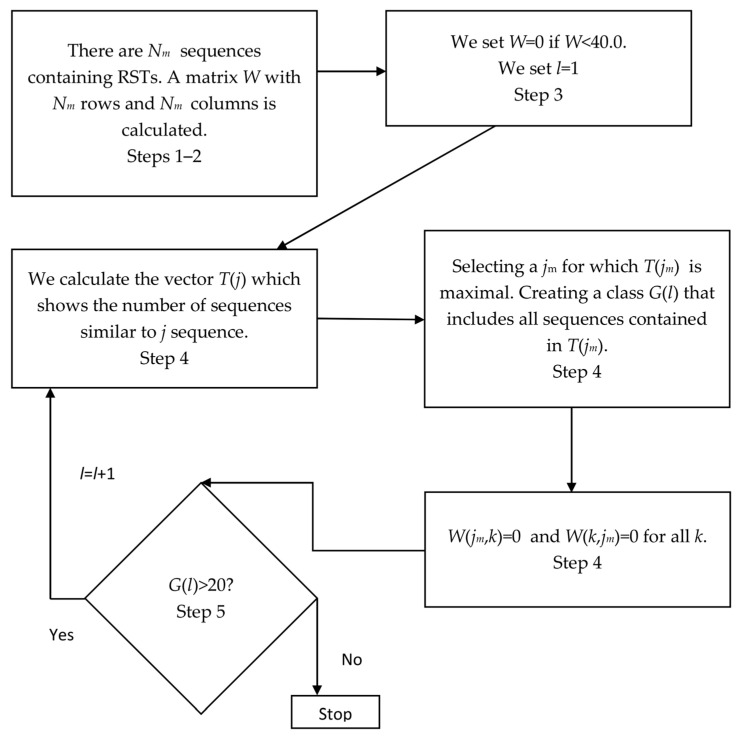
A graphical diagram of the Algorithm 3 for the classifying of regions enriched with RCTs in the bacterial genome is showm in this figure. A detailed description of the Algorithm 3 is shown in Section 4.4.

I also classified the RCT-enriched regions found in the bacterial genome in order to see which triplets occurred in these regions as reverse complementary. To do this, I used the Algorithm 3. The graphical diagram of the algorithm is shown in Figure 6.

**Algorithm 3.** An algorithm for classification of regions enriched with RCTs in the bacterial genome is shown hereStep 1.Let us have *N_m_* regions containing RCTs in the bacterial genome and calculate $x_{T}^{k}(i)$ и $x_{MT}^{k}(i)$ for each of them as described in Section 4.1. Since I were classifying the already found sequences, it could be assumed that for them *k* = 0, i.e., triplets were determined from the first base of the sequence. Then, for each sequence I calculated sum $Xi^{2}=\sum_{i=1}^{32}{\left(x_{T}^{0}\right)}^{2}+\sum_{i=1}^{32}{\left(x_{MT}^{0}\right)}^{2}$ and normalized *Xi*^2^ to 100.0 in order to exclude the influence of the sequence length on $x_{T}^{0}(i)$ and $x_{MT}^{0}(i)$. For this, I introduced correction factor *sk* = (100.0/*Xi*^2^)^0.5^ and then used $y_{T}(i)=sk\times x_{T}^{0}(i)$ and $y_{MT}(i)=sk\times x_{MT}^{0}(i)$ for classification.Step 2Matrix *W*(*N_m_*, *N_m_*) was filled without filling in the main diagonal. The elements of the matrix were sums(5)$$W(j,k)=\sum_{i=1}^{32}y_{T}^{j}(i)y_{T}^{k}(i)+\sum_{i=1}^{32}y_{MT}^{j}(i)y_{MT}^{k}(i)$$ where indices *j* (from 1 to *N_m_*) and *k* (from 1 to *N_m_*) corresponded to the two sequences enriched with RCTs.Step 3I set threshold *W*_0_ = 40.0; for all lesser values, *W* = 0.0 and *l* was taken as 1.Step 4For each *j*, I calculated sum *T*(*j*) for all *k* = 1, …, *N_m_*, where *T*(*j*) = *T*(*j*) + 1 only if *W*(*j*,*k*) ≠ 0.0; here, *T*(*j*) shows the number of sequences that are similar to sequence *j*, provided that *W* ≥ 40.0. I selected such *j_m_* where *T*(*j_m_*) would have the greatest value. The set of all *k* for which *W*(*j_m_*,*k*) ≠ 0.0 would form the class of sequences *G*(*l*) enriched with RCTs; sequence numbers would be recorded in *G*(*l*). Then, I set all rows and columns of matrix *W*(*j*,*k*), whose numbers were included in set *G*(*l*), equal to zero.Step 5If *G*(*l*) ≥ 20, then *l* = *l* + 1, and I proceeded to step 4. If *G*(*l*) < 20, then I stopped creating classes. As a result, I obtained *l* classes, numbers *j_m_* indicating the number of the sequence around which the class was formed, and numbers of sequences included in the class, which are recorded in set *G*.

**Figure 7:**
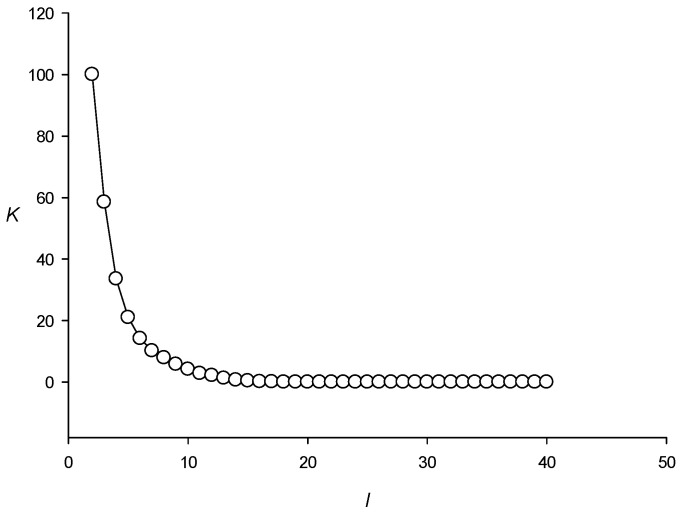
Distribution of classes according to the number of sequences included. Here, *l* is the threshold value for the number of sequences in a class, and *K* is the number of classes with the number of sequences equal to *l* (Section 4.4, step 4).

The threshold of 20 was chosen based on grouping the RCT-enriched regions, provided that all $x_{T}^{k}(i)$ and $x_{MT}^{k}(i)$ (step 1) were randomly shuffled. In total, I created 100 sets, each containing randomly shuffled $x_{T}^{k}(i)$ and $x_{MT}^{k}(i)$ for each of the *N_m_* regions found in the *E. coli* genome. Figure 7 shows the distribution of the number of classes created depending on threshold *l* for the 100 sets; obviously, at *l* ≤ 1 100 classes are created. The results indicate that it was not possible to obtain a single class with more than 20 RCT-enriched regions, which means that in the bacterial genome the number of classes formed purely at random does not exceed 1%.

I also created matrix *MM*(3,4) for each class, which is the sum of matrices *M*(3,4) calculated for each sequence belonging to the class (Section 4.2). For matrix *MM*, I calculated

(6) $$x(i)=\sum_{j=1}^{4}mm(i,j)$$

(7) $$y(j)=\sum_{i=1}^{3}mm(i,j)$$

(8) $$L=\sum_{i=1}^{3}\sum_{j=1}^{4}mm(i,j)$$

Next, I calculated matrix *B*(*i*,*j*) = *x*(*i*)*y*(*j*)/*L* and *P*(*i*,*j*) = *x*(*i*)*y*(*j*)/((*L*)^2^); the variance of the number of bases in cell *mm*(*i*,*j*) of the matrix was found using formula *D*(*i*,*j*) = *LP*(*i*,*j*)(1 − *P*(*i*,*j*)). Then, I obtained matrix

*A*(*i*,*j*) = (*mm*(*i*,*j*) − *b*(*i*,*j*))/(*D*(*i*,*j*)^0.5^) (9)

I also calculated $x_{T}^{0}(i)$ and $x_{MT}^{0}(i)$ for each created class. To do this, I determined *f*(*i*) and probabilities *p*(*i*) = *f*(*i*)/*L* for each sequence in the class, where *f*(*i*) is the frequency of bases a, t, c, g and *L* is the total length of all sequences in the class. The probability of detecting a triplet from set *T* in sequence *S* was calculated as *P_T_*(*t*) = *p*(*i*)*p*(*j*)*p*(*k*), where *i*, *j*, and *k* are the DNA bases in the first, second, and third positions of the triplet, respectively. Then, the average number of triplets *t* in all sequences of the class was equal to variance *D*(*t*) = (*L*/3)*P_T_*(*t*)(1 − *P_T_*(*t*)) and

(10) $$x_{T}(i)=(n(t)-\bar{n}(t))/\sqrt{D(t)}$$

where *n*(*t*) is the number of triplets *t* in all sequences from the class. *x_MT_*(*i*) for set *MT* was calculated in the same way as *x_M_*(*i*).

As a result, I obtained:

*RRR*(*i*) = *x_M_*(*i*) *x_MT_*(*i*) (11)

This parameter made it possible to estimate the contribution of each triplet and its mirror copy to the formation of a sequence with RCTs.

### 4.5. Creation of RCT-Enriched Sequence Groups for the Genomes of 42 Bacteria

Then, I created groups of matrices *MM* found for 42 bacterial species; the set of these matrices was denoted as *BM*. Each matrix *MM* was calculated for the central sequence of a class that contained RCT-enriched sequences existing in the bacterial genome (Section 4.4). To create matrix groups, I first calculated the maximum value of sum *L*_2_ (Section 4.4) for all *MM* matrices of all bacteria, which turned out to be 4.12 × 10^5^; the purpose of this step was to exclude the influence of *MM* matrix sizes on group creation. Each element of matrix *MM* was recalculated as *mm*(*i*,*j*) = 4.12 × 10^5^*mm*(*i*,*j*)/*L*_2_, and for each of the recalculated matrices I determined matrix *A*(*i*,*j*) using Formulas (6)–(11). *A*(*i*,*j*) shows the bases in triplet positions that are expected to be more frequent than in a randomly shuffled sequence; the set of *A*(*i*,*j*) matrices was denoted as *BA*.

Thus, each matrix *MM* from set *BM* corresponded to matrix *A* from set *BA*, which means that the classification of matrices *MM* can be performed using that of matrices *A*. To do this, I employed the similarity measure of two matrices, *A^k^*(*i*,*j*) and *A^l^*(*i*,*j*):

(12) $$R(k,l)=\sum_{i=1,}^{3}\sum_{j=1}^{4}A^{k}(i,j)A^{l}(i,j)$$

where *k* and *l* vary from 1 to 195 which is a total number of classes of RCT-enriched sequences in bacterial genomes created in this work.

For each matrix *A* from set *BA*, I created matrix *Arand* by randomly shuffling the elements of matrix *A* and denoted this set of matrices as *BArand*. I also introduced matrix *Rmax*(*k*,*l*) = 0 for all *k* and *l*. Then, I compared matrices *Ak*(*i*,*j*) and *Al*(*i*,*j*) using Formula (5) and stored the resulting number *R*(*k*,*l*) in matrix *Rmax*(*k*,*l*), if *R*(*k*,*l*) > *Rmax*(*k*,*l*). These calculations were performed for all *k* and *l*. After that, I generated a new set *BArand* and again compared the two matrices *Ak*(*i*,*j*) and *Al*(*i*,*j*) using Formula (5). This cycle was repeated 100 times. As a result, I obtained number *Rmax*(*k*,*l*) for each pair of matrices *k* and *l* from set *BA*, which was recorded in the group_r100.txt file.

Next, I grouped matrices from set *BA* using the Algorithm 4.
**Algorithm 4.** Here is the algorithm that I used to create of RCT-enriched sequence groups for the genomes of 42 bacteriaStep 1Let *t* = 0. I filled vector *Ri*(*k*), where the number of matrices with *R*(*k*,*l*) > *Rmax*(*k*,*l*) (l from 1 to 195) was counted for each matrix *k* from set *BA* and denoted as *N*(*k*). The numbers of these matrices were recorded in matrix *Nom*(*k*,*i*), *i* = 1, …, *N*(*k*).Step 2I selected the value of *k* for which *N*(*k*) was maximal and set *t* = *t* + 1; thus, the first group of matrices *Gr*(*t*) was created.Step 3I took *N*(*k*) = 0 and excluded from matrix *Nom* all matrix numbers listed in *Nom*(*k*,*i*), *i* = 1, …, *N*(*k*). The exclusion was performed according to index *k* and the numbers contained in *Nom*(*k*,*i*) for all *i*.Step 4If *Gr*(*t*) > 10.0, I proceeded to step 1, and if *Gr*(*t*) ≤ 10.0, the creation of classes was stopped. For each class, I obtained the number of central matrix *A* and a set of matrices that belonged to this group. I also recorded the cells in the central matrix with the values ≥ 3.0. The condition *Gr*(*t*) ≤ 10.0 was due to the fact that if the cells within matrices *A^k^*(*i*,*j*) and *A^l^*(*i*,*j*) were randomly mixed, the size of the classes *Gr*(*t*) did not exceed 6. Therefore, the classification performed can be considered non-random.

## 5. Conclusions

I developed a method to search bacterial genomes for sequences enriched in reverse complementary triplets (RCTs). This method makes it possible to find RCTs in the presence of strongly expressed triplet periodicity characteristic for coding sequences. I analyzed 42 genomes of bacteria from all phyla and found that the number of RCT-enriched sequences ranged from hundreds to thousands per genome depending on the genome size. The average length of the region with RCTs was about 850 DNA bases. The most statistically significant inversion symmetry (IS) pattern was the enrichment of triplet positions 1, 2, and 3 with bases {A,G}, {A,T}, and {T,C}, respectively. When the sequence was rotated 180 degrees and the bases were replaced with complementary ones (IS), this enrichment of triplet positions was preserved. I suggest that the emergence of IS may be a result of evolutionary processes such as inversions, transpositions, and recombinations.

## Figures and Tables

**Table 1:** Number of regions (*Nm*) enriched with RCTs in the *E. coli* genome (*N*_0_ is the number of false positives and *V*_0_ is the threshold level).

*V*_0_	3.0	3.5	4.0	4.5	5.0	5.5	6.0
*N_m_*	3034	2617	2180	1726	1348	1040	772
*N*_0_	1580	676	258	87	35	13	5

**Table 2:** The number of sequences with RCTs in the genomes of bacteria from different phyla for *V*_0_ = 4.5.

*N*	Phyla	Species	*N_m_*	*N*_0_	*p* (%)	Genome Size, 10^6^ bp
1	Acidobacteriota	*Acidobacterium capsulatum*	1055	92	8.7	4.1
2	Actinomycetota	*Actinomyces israelii*	308	58	18.8	2.6
3	Aquificota	*Thermosulfidibacter takaii*	614	35	5.7	1.8
4	Armatimonadota	*Chthonomonas calidirosea*	302	56	18.5	3.4
5	Atribacterota	*Candidatus Atribacteria bacterium*	430	65	15.1	2.7
6	Bacillota	*Bacillus anthracis*	351	150	42.7	5.2
7	Bacteroidota	*Bacteroides fragilis*	624	100	16.0	3.5
8	Balneolota	*Balneola sp.*	854	86	10.0	4.0
9	Bdellovibrionota	*Bdellovibrio bacteriovorus*	1337	83	6.2	3.8
10	Caldisericota	*Candidatus poribacteria bacterium*	357	126	35.3	4.9
11	Calditrichota	*Calditrichaeota bacterium*	1509	78	5.2	3.6
12	Campylobacterota	*Helicobacter pylori*	1042	35	3.3	1.6
13	Chlamydiota	*Chlamydia muridarum*	316	28	8.9	1.1
14	Chlorobiota	*Chlorobium chlorochromatii*	1107	58	5.2	2.6
15	Chloroflexota	*Dehalogenimonas lykanthroporepellens*	606	43	7.1	1.7
16	Chrysiogenota	*Desulfurispirillum indicum*	1136	62	5.4	3.0
17	Coprothermobacterota	*Coprothermobacter proteolyticus*	267	26	9.7	1.5
18	Deferribacterota	*Calditerrivibrio nitroreducen*	463	58	12.3	2.3
19	Deinococcota	*Truepera radiovictrix*	658	64	9.7	3.3
20	Dictyoglomota	*Dictyoglomus thermophilum*	725	56	7.7	2.0
21	Elusimicrobiota	*Elusimicrobium minutum*	1107	42	3.8	1.6
22	Fibrobacterota	*Fibrobacter succinogenes*	832	66	7.9	3.6
23	Fusobacteriota	*Fusobacterium necrogenes*	369	40	10.8	2.0
24	Gemmatimonadota	*Gemmatimonas aurantiaca*	2120	98	4.6	4.7
25	Ignavibacteriota	*Ignavibacterium album*	1138	105	9.2	3.7
26	Kiritimatiellota	*Kiritimatiellaceae_bacterium*	207	39	18.8	1.7
27	Lentisphaerota	*Victivallis vadensis*	2399	145	6.0	5.2
28	Mycoplasmatota	*Spiroplasma poulsonii*	418	72	17.2	2.3
29	Myxococcota	*Lentisphaerae bacterium*	1888	112	5.9	4.3
30	Nitrososphaerota	*Nitrosopumilus_maritimus*	792	46	5.8	1.6
31	Nitrospinota	*Nitrospina gracilis_*	1105	59	5.3	3.1
32	Nitrospirota	*Nitrospira moscoviensis*	1065	102	9.6	4.6
33	Planctomycetota	*Gemmata obscuriglobus*	1296	208	16.0	9.0
34	Pseudomonadota	*Salmonella enterica*	2090	75	3.5	5.0
35	Rhodothermota	*Rhodothermaceae bacterium*	455	98	21.5	4.7
36	Spirochaetota	*Leptospira interrogans*	2579	169	6.5	5.1
37	Synergistota	*Anaerobaculum hydrogeniforman*	2570	43	1.6	2.1
38	Termoproteota	*Crenarchaeota_archaeon*	471	72	15.2	3.5
39	Thermodesulfobacteriota	*Sulfolobus acidocaldarius*	256	45	17.5	2.3
40	Thermomicrobia	*Thermomicrobium roseum*	1513	62	4.1	3.0
41	Thermotogota	*Petrotoga mobilis*	269	47	17.4	1.8
42	Verrucomicrobiota	*Chlamydia trachomatis*	521	72	13.8	2.4

**Table 3:** Classes of sequences with RCTs found in the *E. coli* genome.

Class Number	Number of Sequences in the Class	Cells in Matrix *A* (Section 4.5) That Are Greater than 3.0 (Matrix *A* Values Shown in Brackets)
1	1261	1/A (6.9), 1/C (10.4), 1/G (29.3), 2/A (41.7), 2/T (43.0), 3/T (5.5), 3/C (31.3), 3/G (10.2)
2	116	1/A (7.1), 1/G (49.8), 2/A (34.9), 2/T (28.9), 3/T (17.4), 3/C (36.7)
3	70	1/T (43.2), 1/C (3.3), 2/C (23.9), 2/G (22.6), 3/A (36.0), 3/G (8.2)
4	50	1/C (12.4), 1/G (9.9), 2/A (21.7), 2/T (10.1), 3/C (6.1)
5	31	1/C (16.0), 1/G (28.7), 2/A (41.3), 2/T (29.0), 3/T (14.2), 3/C (12.4), 3/G (9.6)
6	21	1/G (16.1), 2/A (19.7), 2/T (7.9), 3/C (20.4)

**Table 4:** Groups of sequences with RCT created for 195 classes from 42 bacteria.

Group Number	Central Class Number	Number of Classes in the Group	Cells in Matrix *A* (Section 4.5) Greater than 3.0 (Matrix *A* Values Shown in Brackets)
1	153	77	1/A (6.7), 1/G (45.4), 2/A (46.6), 2/T (46.9), 3/T (7.3), 3/C (39.5), 3/G (3.7)
2	43	36	1/T (44.5), 2/C (38.1), 2/G (48.4), 3/A (38.9)
3	74	29	1/A (6.5), 1/G (26.5), 2/C (14.6), 3/T (8.8), 3/C (7.2)
4	154	15	1/T (33.5), 1/C (11.3), 2/C (18.1), 2/G (18.7), 3/A (31.9), 3/G (12.4)
5	166	11	1/C (13.4), 1/G (6.5), 2/A (9.0), 2/T (12.2), 3/G (14.1)
6	108	10	1/A (13.2), 1/G (18.4), 3/T (5.6), 3/C (28.2)

**Table 5:** *SR*^0^(*L*,4) calculated for several lengths *L* of three bacterial genomes with different A + T composition.

*L*	300	390	480	570	660	750	840	930	1020	1100
*E. coli* A + T = 50%	60.0	80.0	93.6	102.8	110.7	117.0	123.9	125.9	136.6	143.1
*Truepera radiovictrix* A + T = 32%	73.2	89.7	100.6	109.2	116.1	123.1	128.2	135.5	142.3	148.3
*Spiroplasma poulsonii* A + T = 74%	59.0	74.4	85.5	96.3	104.4	110.8	120.2	126.1	132.0	140.2

**Table 6:** *D*^0^(*L*,4) calculated for several lengths *L* of three bacterial genomes with different A + T composition.

*L*	300	390	480	570	660	750	840	930	1020	1100
*E. coli* A + T = 50%	24.0	29.6	33.6	34.4	37.8	39.6	40.2	41.5	42.8	45.0
*T. radiovictrix* A + T = 32%	32.0	37.6	40.8	39.6	41.0	43.4	42.6	45.8	46.6	46.8
*S. poulsonii* A + T = 74%	25.0	31.8	32.6	34.6	38.0	38.7	40.2	43.0	44.0	45.2

**Table 7:** *Z* levels of TP, for which *SR^k^*(*L*,*N*) and *D^k^*(*L*,*N*) were calculated separately.

Interval Number *N*	1	2	3	4	5	6	7
*Z* range	−5–2	2–4	4–6	6–10	10–14	14–20	20–100

## Data Availability

All received data are in Supplementary Materials. The calculations were done on the website http://victoria.biengi.ac.ru/mct (accessed on 20 February 2026).

## Supplementary Materials

The following supporting information can be downloaded at: https://www.mdpi.com/article/10.3390/ijms27052301/s1.
