# Supplementary material for: Regions Enriched with Reverse Complement Triplets in Bacterial Genomes
Source: Int J Mol Sci. 2026 Feb 28;27(5):2301. doi: 10.3390/ijms27052301 (PMC12985086; doi:10.3390/ijms27052301)
Supplement: Supplementary file 1 [file ijms-27-02301-s001.zip › read.me.pdf]

About MCT method

We have developed a mathematical method to search for DNA regions that are significantly enriched in reverse complement triplets (RCTs) and are located in sequences with strongly expressed triplet periodicity (TP). The method makes it possible to exclude the influence of TP on the number of RCTs. To search for RCTs, we used the difference between triplet frequencies and their expected number, which was determined by taking into account the TP of the analyzed region.

About MCT tool (http://victoria.biengi.ac.ru/mct)

Significance level - number of the identified sequences with RCTs depending on threshold. You must specify this level as the normal distribution argument. By default, we use a level of 4.5. If you want to reduce the number of false positives, you should choose a higher level.
The program creates two output files. The first is named selection.txt. It contains the results of studying the input sequence. The second file is selection_r.txt. It contains the results of studying the random sequence

a. selection.txt - contains the results of a search for DNA regions containing mirror-symmetric triplets in greater numbers than expected.
kok - the number of the DNA region found
KK - the beginning of the region
LL - the end of the region
Z - the level of statistical significance in the arguments of the normal distribution
DL - the length of the found region
A=== and B=== represent mirror-complementary triplets. Here 1 is A; 2 is T; 3 is C; 4 is G
X1== is the statistical significance of triplets in the arguments of the normal distribution shown in the "A===" field.
X2== is the statistical significance of triplets in the arguments of the normal distribution shown in the "B===" field.
KKK= - the product of X1 and X2 for each pair of mirror-complementary triplets
CD1 - the number of each triplet shown in the X1=== field
CD2 - the number of each triplet shown in the X2=== field
The last line contains the found sequence.

b. selection_r.txt - contains the results for the DNA regions where the studied sequence was scrambled by codon positions. For this, three subsequences were created. The first contained bases in the first position of the triplets, the second contained bases located in the second position of the teriplet, and the third contained bases located in the third position of the triplet. Then, all three sequences were independently randomly scrambled and inserted into the corresponding triplet positions. This shuffling method preserved the statistical properties of the analyzed sequence, while randomizing the content of the mirror-complementary triplets. This file can be considered to contain the detected false positives.
